# ‘It felt like surveillance, not support’: understanding barriers to enrolling older adults in an energy-linked digital health study during pandemic recovery and the cost-of-living crisis

**DOI:** 10.1186/s12877-026-07665-7

**Published:** 2026-05-28

**Authors:** Bertha Ochieng, Victoria Hall, Rebecca Ochieng, Jane Amedzro, Richard Wong

**Affiliations:** 1 Faculty of Health & Life Sciences, Centre for Primary Care Research De Montfort University, The Gateway, Leicester, LE1 9BH United Kingdom; 2 Faculty of Health & Life Sciences, De Montfort University, The Gateway, Leicester, LE1 9BH United Kingdom; 3 Willows Health, 184 Coleman Road, Leicester, LE5 4LJ United Kingdom

**Keywords:** Older adults, Digital health, Recruitment barriers, Privacy mistrust, COVID-19, Cost-of-living crisis

## Abstract

**Background:**

While digital health tools are now routinely encouraged across UK health and social care, older adults — particularly those managing multiple long-term conditions — continue to be under-represented in community-based research. Exploring why engagement falls short is essential for improving involvement in technology-supported ageing initiatives.

**Methods:**

This paper reports findings from a qualitative study exploring barriers to participation in a digital health research project involving older adults. The qualitative work included a workshop with recruitment staff (*n* = 4), and discussions with key gatekeepers — community organisation leads (*n* = 4) and carers of older adults (*n* = 2). Data were analysed using a Framework-guided approach. The qualitative component was embedded within a feasibility study designed to examine a smart-meter mobile application intended to monitor everyday electricity-use patterns among housebound adults aged ≥ 65, with the aim of detecting shifts in daily routines that may signal changes in health. Recruitment took place across the East Midlands between 2021 and 2022, during a period shaped by COVID-19 recovery and the national cost-of-living crisis, but yielded minimal uptake and did not proceed to data collection.

**Results:**

Analysis of the qualitative data identified four interrelated influences shaping decisions not to participate: misunderstandings about the purpose and function of the technology; heightened mistrust and privacy concerns in the context of rising energy prices; limited motivation to engage in research alongside a desire to avoid being associated with “frailty”; and the broader environment of pandemic fatigue and increased exposure to technology-related scams. Some older adults expressed concerns that their energy data might be misused — for billing or by criminals — and several avoided telephone contact altogether, describing the phone as no longer a safe place.

**Conclusions:**

Involving older adults in digital-health studies that draw on domestic energy data must account for the broader social and economic environment. Financial strain, digital fraud, and pandemic-related mistrust all influenced disengagement. Early community involvement, co-design, and opportunities to discuss or engage directly with the technology may support more confident and informed participation in future research.

**Supplementary Information:**

The online version contains supplementary material available at 10.1186/s12877-026-07665-7.

## Background

While digital health tools are now routinely promoted across UK health and social care, older adults — particularly those managing multiple long-term conditions — remain under-represented in community-based research [1]. This gap is not simply a matter of access, but also reflects issues of trust, perceived relevance, and how research participation itself is understood [2]. Addressing these barriers is important if technology-supported approaches to ageing are to be more inclusive.

Recruitment to digital health studies can be particularly challenging [3]. Approaches that rely on passive data collection, remote monitoring, or unfamiliar forms of data are not always easy to explain and may be interpreted as intrusive or unnecessary [4]. These difficulties tend to be more pronounced among groups who are already less well served by healthcare systems, including those with limited digital confidence, financial pressures, or weaker engagement with formal services [5].

Examining why recruitment efforts fall short—and how potential participants make sense of research invitations—is therefore important. Although previous studies have identified barriers such as digital exclusion, privacy concerns, and limited perceived benefit [6], there remains limited empirical work exploring how these issues experienced and negotiated in practice, particularly in periods shaped by wider social and economic disruption. These challenges also need to be understood within the wider shift towards adoption of digital approaches in health and social care, which shapes both how their application within the care of older adults are designed and how older adults decision to participate in digital health research is negotiated.

Large-scale data and the digital traces people leave through everyday activities are increasingly used to understand patterns in health, wellbeing, and social life. These forms of “everyday data” are becoming central to efforts across health and social care to anticipate need and shape more responsive support models [7]. Technologies now deployed in healthcare span implantable devices, laboratory-based diagnostics, mobile applications, and a range of software-enabled monitoring tools. Although older adults have traditionally adopted new technologies at a slower pace [8], digital engagement accelerated across all age groups during the COVID-19 period [9, 10].

Against this backdrop, the UK’s ageing population and the rising complexity of later-life health needs highlight the importance of effective home-based support. Older adults living with multiple long-term conditions and varying levels of frailty account for a significant proportion of health and care use [11], and projections indicate that the number of people with multimorbidity will continue to increase substantially by 2035 [12]. This trend places added pressure on both primary and secondary care services [13]. Policy documents such as the NHS Long Term Plan emphasise the role of digital approaches in primary and outpatient settings to help reduce avoidable hospital attendances [14]. Early detection of functional decline is particularly valuable, and devices including accelerometer-based wearables are already being explored to monitor mobility and gait [15].

For many older adults, remaining at home is closely tied to dignity, autonomy, and a sense of identity. Technologies designed to support independent living are therefore more likely to be accepted when they align with individuals’ preferences, privacy expectations, and levels of digital confidence. One emerging approach within this landscape involves the use of passive, home-based data to infer changes in daily routines and potential health decline.

Against this backdrop, we designed a community-based study to explore whether a remote monitoring app using smart-meter data could help identify day-to-day activity patterns among older adults who rarely leave their homes. The approach relied on analysing household electricity-use data to establish each person’s usual routine and identify deviations that might indicate changes in health or wellbeing. Following an initial baseline period, deviations in routine were intended to trigger notifications, enabling carers to check on the individual and, where appropriate, prompt timely intervention. However, the implementation and reception of such approaches cannot be separated from the wider social and economic conditions in which they are introduced.

Recruitment occurred during 2021–2022 — an exceptional period marked by two overlapping crises. First, the lingering effects of COVID-19 left many older adults socially isolated, distrustful of unsolicited contact, and wary of health-related technology. Second, the UK entered a severe cost-of-living crisis, with steep rises in food, rent, and especially energy prices [16, 17]. Lower-income households, including many older pensioners, faced fuel poverty and made health-compromising cutbacks such as reducing their use of heating [18]. Financial anxiety was accompanied by a surge in digital and energy-related scams, including phishing messages and fraudulent energy-related offers [19–21].

These combined pressures fostered an atmosphere of mistrust. Older adults were repeatedly advised to avoid unknown phone numbers or unsolicited contact, making traditional research recruitment channels — including telephone calls, email invitations, and online advertisements — increasingly ineffective. This climate shaped the recruitment dynamics of the study, and recruitment proved particularly challenging. In response, a multi-pronged strategy was implemented, including:

- Distribution of flyers in community centres and primary care practices
- Online advertisements through older-adult activity networks
- Direct telephone outreach supported by health and social care practitioners

Potential participants were identified through community organisations and primary care networks. Initial contact was usually made by community-based gatekeepers or health and social care workers, who introduced the study to individuals within their networks. Direct contact with the research team occurred only where individuals expressed an interest in participation. Recruitment therefore largely relied on intermediary-led engagement rather than direct researcher approach, particularly during the COVID-19 recovery period. Across the recruitment period, approximately 150 potential participants were approached. Despite some initial expressions of interest, only four individuals proceeded to enrolment, and the majority of those approached declined participation or did not progress further. This study therefore aimed to explore the barriers, perceptions, and contextual factors shaping non-participation in a digital health study involving domestic energy data, and to identify implications for improving recruitment of under-represented groups in similar research.

## Methods

### Study design

Due to low recruitment, the research team undertook a qualitative evaluation to examine why older adults declined participation.

### Participants and data collection

Data was collected through three complementary sources:

1. Workshop with Health and Social Care Workers (HSCWs) who were directly involved with the recruitment process (*n* = 4).
2. Interviews with community organisation leads (COL) who supported the recruitment drive (*n* = 4).
3. Interviews with carers of potential participants who had declined participation (*n* = 2).

The semi-structured qualitative interview and workshop guide was developed specifically for this study and has not been previously published. It was developed a priori and informed by existing literature on digital health use among older adults and barriers to research participation. It was designed to capture the perspectives of recruiters and carers in relation to the study aims and is provided as a supplementary file. Each session explored three broad areas:

- Barriers to recruitment
- Reasons for non-participation
- Alternative recruitment approaches

Apart from the HSCW workshop, all interviews were conducted individually and lasted between 30 and 60 min. The HSCW workshop lasted about 90 min. During the interviews and workshop, the participants were encouraged to reflect freely on practical and ethical considerations surrounding recruitment. All discussions were held online or by telephone between September 2022 and March 2023, audio-recorded with consent, and transcribed verbatim. Field notes were recorded during and immediately after interviews and workshop to support and complement the recorded data, including noting practical details such as participant presence and key points during discussions.

### Analytical approach

We analysed the qualitative material using an approach informed by Framework methodology [22, 23]. Analysis began with familiarisation, with researchers reading transcripts and field notes in full. Two researchers undertook initial line-by-line open coding of a subset of transcripts to identify key concepts and recurrent issues. These initial codes were discussed within the research team and used to develop a provisional analytic framework, comprising a set of defined categories linked both to patterns emerging from the data and to the study aims.

The framework was subsequently applied across the full dataset, with data indexed and charted into a matrix organised by individual participant and thematic category. This matrix supported comparison of patterns both within and across participants and data sources, providing a structured and transparent basis for analysis. Coding and the framework were refined iteratively as the analysis progressed. Coded data and emerging categories were reviewed by a third researcher, who contributed to the interpretation and refinement of the analytic framework. The developing analysis was then discussed with the lead researcher, who provided final oversight and interpretive input. Emerging patterns were reviewed in regular team discussions, allowing categories to be consolidated and developed into higher-order themes. Final themes were generated through an interpretive process that considered both convergence and variation across the dataset. This approach also supported interpretation by enabling the findings to be situated within wider discussions of digital inclusion and ageing.

Although data was gathered from HSCW, COL gatekeepers and the carers of two potential older adults who were housebound, their accounts were analysed collectively rather than comparatively. This approach was intentional and grounded in both the study’s aim and the nature of the dataset. The purpose was not to contrast perspectives but to understand the shared and intersecting barriers that contributed to recruitment failure. Across all groups, the themes demonstrated a high degree of consistency and overlap: concerns about surveillance, privacy, and energy costs were echoed almost verbatim by the HSCW, COL gatekeepers and carers alike. Treating these voices together therefore provided a more coherent picture of the relational and contextual dynamics shaping recruitment, rather than fragmenting a small dataset into sub-group analyses. In this sense, the inclusion of multiple yet overlapping perspectives was a strength, allowing for a richer triangulation of barriers and yielding a more holistic understanding of why recruitment was unsuccessful in this particular social and economic context. The reporting of this qualitative study was guided by the Standards for Reporting Qualitative Research (SRQR) [24], supporting clarity in the description of methods and analysis.

## Results

The framework analysis generated four overarching and inter-related themes that captured the core barriers to recruitment:

1. Misperceptions about energy-use monitoring and its link to daily living activities.
2. Mistrust and privacy concerns — including the influence of family, friends, and carers.
3. Apathy, autonomy and resistance to research participation labelled.
4. Recruitment within the social context of COVID-19 recovery and the UK cost-of-living crisis.

### Misperceptions about energy-use monitoring and its link to daily living activities

Potential participants struggled to understand how electricity-use data could relate to health or wellbeing. Recruiters repeatedly described confusion and disbelief when explaining how smart-meter data might help identify changes in daily activities:

*They had a smart meter*,* but they couldn’t see how that would relate to their health.* (Fatima, COL)

*The minute I start talking about electricity or something to do with an app*,* it threw them off a little bit. It was like*,* ‘Why are you calling from my doctor’s surgery?’* (Eve, COL).

Recruiters often described low technology literacy as making the concept difficult for those approached to understand:

*Yeah*,* they just couldn’t get their head around smart meters monitoring health. I couldn’t get into the conversation enough even to really explain it.* (Fatima, COL)

Some rejected the idea outright as *too technical* or *irrelevant*. In HSCWs discussions, recruiters reflected that several older adults interpreted the app as replacing, rather than supporting, human care:

*It felt like somebody was taking care of them via an app… that app became the carer.* (HSCW)

Ben, a carer, reported that some older adults expressed their fear that their independence might be obstructed if they took part in the study and viewed the technology as being a risk to continuing their current lifestyle, by stating:

*One day I might pop out to the shops or go bingo… I’ve not been home for eight hours — is someone gonna say*,* why are you not home?*

While Edward, another carer, also reported and indicated that some older adults would express their concerns by stating that:

*If I popped to the shop and then decide to bring a friend back*,* you’ve picked up electricity usage again.*

Older adults perceived the technology as intrusive rather than enabling. Recruiters later recognised that the study had been unintentionally framed as monitoring rather than self-management by some potential participants and their next of kin/carer, triggering fears of surveillance and loss of independence.

### Mistrust and privacy concerns — including the influence of family, friends, and carers

Mistrust was the most reported barrier, cutting across all participants who contributed to the reflection interviews and consultations. They indicated that older adults or their next of kin described deep unease about being observed at home and uncertainty about who could access their data:

*There was something about it he didn’t trust. I couldn’t explain why*. (Edward, carer)

*One said it was Big-Brotherly.* (Fatima, COL)

*It was definitely an invasion of privacy.* (Eve, COL)

All participants indicated and highlighted concerns expressed to them by most older adults during the recruitment phase that the app was intrusive to their home life and these were expressed as follows:

*I don’t want anyone to realise I was in the bathroom or toilet.* (Ben, carer)

Others accounted how older adults feared that data might be used by criminals and indicated that older adults reported:

*You’re asking me about my electricity*,* which means you can keep an eye on when I’ve gone to bed. What if I get burgled in the hours you’ve noticed I’ve not moved?* (Abdul, COL)

Questions about data security and ownership were commonly encountered by recruiters:

*He didn’t understand what’s going on — who is dealing with it*,* and where the information will be sent to.* (HSCW)

Even calls from GP practice numbers, which originated from the HSCWs who were based in primary care settings, were met with suspicion:

*They accused me of lying and said I’m from an electricity company.* (HSCW)

The HSCW indicated that older adults found it implausible that primary care practices could be connected to energy-use monitoring:

*As soon as they hear someone’s gonna come home to look at the smart meter*,* they don’t believe it’s from the practice*. (HSCW)

Amid record energy prices, some believed the study was a disguised attempt to check their consumption. Eve, a COL, indicated that older adults would state:

*You’re trying to get my meter readings so that you can charge me the accurate price.* (Eve, COL)

Others associated recruitment with commercial sales. As one COL summarised:

*There’s a substantial degree of mistrust of outsiders asking questions about personal or financial situations*,* and of devices in the home that might be gathering that information.* (Ezra, COL)

Recruiters’ reassurances that the study incurred no cost often failed:

*We’re not here to detect your bills. Nothing will be costing you. But they think*,* ‘OK*,* you want to see how much data I’m using.’* (Eve, COL).

Economic hardship compounded these fears:

*She’s really struggling with bills. She turns her lights off and avoids using the kettle because it’s too expensive.* (Ben, carer)

Mistrust thus stemmed from multiple sources — data privacy, financial insecurity, and scepticism towards being part of the research and institutions — which collectively contributed to non-participation.

### Apathy, autonomy and resistance to perceived research participation identity

Older adults responded to the research invitation in varied ways, ranging from passive disengagement to active resistance. For some, the invitation generated apathy or low motivation, often expressed through brief interactions in which individuals showed little.

inclination to explore the purpose of the study:

*No interest — it was just*,* no*,* I’m not interested. They just wanted to get me off the phone.* (Fatima, COL)

This apathy appeared shaped by pandemic fatigue, competing priorities, and a perception that the study offered limited personal benefit in a time of instability. Research was seen not as supportive but as an additional demand, and for many, the immediate pressures of health, finances, and everyday coping outweighed any potential value of participation.

Alongside this, a clear strand of identity-based resistance emerged. Some older adults rejected what they perceived as implicit assumptions of vulnerability or frailty, distancing themselves from narratives that conflicted with their sense of independence:

*I’m not a little old man. Who said that I need support? I’m 92*,* fit and fine. I live on my own — I don’t need their help.* (Ezra, COL)

Here, refusal was not about disinterest but about protecting autonomy and resisting perceived labels that felt misaligned with how they viewed themselves.

Family members also played a role in shaping decisions. Adult children and carers often acted as informal gatekeepers, filtering information or discouraging participation:

*The carers muscled in and said don’t.* (Fatima, COL)

*I’ll speak to my son or daughter and get back to you’ — but they never did.* (Eve, COL)

Some next of kin expressed fears that technology might replace their caregiving role:

*We’re here for you — you don’t need that type of service.* (HSCW)

Limited digital literacy among carers sometimes heightened these concerns, resulting in protective decisions made on behalf of the older adult. In several cases, these family dynamics further complicated recruitment by influencing or overriding the older adult’s own autonomy. The theme reflects how motivation, identity and autonomy intersected within the research invitation process. Rather than simple refusal, decisions were embedded in wider socio-emotional contexts — including the desire to maintain control, avoid unwanted labels, and navigate complex family relationships at a time of heightened uncertainty.

### Recruitment within the social context of COVID-19 and the UK cost-of-living crisis

The social environment shaped every aspect of recruitment. During the COVID-19 recovery period, older adults had become cautious about all unsolicited contact. Recruiters reported that scams had fundamentally changed communication norms:

*The phone is no longer a safe place.* (Fatima, COL)

Other COL also noted the escalated mistrust of online tools that also impacted the reluctance of older adults and their next of kin to participate in the study:

*Smartphone use was patchy*,* and there was widespread mistrust of Zoom*,* fuelled by early stories of security breaches.* (Ezra, COL)

Health and social care workers on the workshop indicated that even verified calls were often ignored:

*Getting an answered phone was challenging — being able to even explain who you were was really difficult.* (HSCW)

One of the HSCW indicated that at times those who did answer demanded extensive verification:

*They make me verify absolutely everything — who they last saw*,* which clinician*,* what day*,* what time.* (HSCW)

Trust in primary care practices had also eroded due to the pandemic and a HSCW recounted a phone discussion with an older adult, who informed him:

*‘I don’t have anything to do with my practice anymore… I can’t even get an appointment.’* (HSCW).

The city’s prolonged lockdown and high infection rates amplified fatigue and isolation. At the same time, the cost-of-living crisis heightened anxiety around any project linked to energy use. This was demonstrated by feedback from an older adult reported by a HSCW as:

*‘You’re trying to get my meter readings so that you can charge me the accurate price.’* (HSCW).

While a COL indicated that a participant advised them

*I turn my lights off… I’ve reduced kettle usage* (Ben, carer).

Some leaders from community organisations noted that any reassurances they provided to older adults regarding the protection of privacy and adherence to GDPR were often inadequate. One leader recounted a conversation with an older adult where she tried to reassure him about these concerns.

*We’re not here to detect your bills. Nothing will be costing you.* (Eve, COL)

While other older adults doubted the system’s functionality and links to how the usage of electricity could determine their activities of daily living in a cost-of-living crisis. A COL indicated that an older adult stated:

*‘I’ve cut back so much; the device won’t pick anything up*.’ (Abdul, COL).

While other older adults were reported as questioning the reliability of the app in monitoring their activities of daily living, and had stated:

*‘How do you know if I’ve had a fall?’* (HSCW).

The combination of pandemic-related distrust, economic stress, and perceived surveillance made engagement emotionally burdensome. As one HSCW reflected, during the workshop

*The study came at the wrong time—people were simply exhausted*,* anxious*,* and protective of their privacy.*

## Discussion

The purpose of this study was to understand why a home-based, technology-supported monitoring initiative for housebound older adults in Leicester did not attract participants during 2021–2022. The analysis indicates that the challenge lay not in the volume of recruitment activity but in a fundamental disconnect between the study’s framing of smart-meter data as a health tool and how older adults, their families, and their support networks interpreted energy data and digital surveillance during this period. While some of the barriers identified here broadly align with those reported in previous research on participation in digital health studies, the findings also illustrate how these issues are encountered in practice. In particular, the study draws on the perspectives of health and social care workers involved in recruitment, alongside community intermediaries—voices that are less frequently represented in this area of research—providing insight into how these barriers are communicated, interpreted, and negotiated during the recruitment process. These accounts indicate that concerns around mistrust, privacy, and perceived relevance were shaped by the wider socio-economic context. The combined pressures of pandemic recovery and the cost-of-living crisis further intensified these concerns, influencing how the study was understood and contributing to low levels of participation.

The first theme illustrates that many older adults struggled to connect an energy-monitoring device with a health-related purpose. Smart meters were widely understood as instruments for billing or energy-efficiency, so their redeployment for health monitoring felt counterintuitive. This produced confusion (*they couldn’t get their head around it*) and, for some, immediate refusal (*this is too technological for me*). Prior research has noted that when the rationale for a digital device is unclear or its benefits accrue over time, older adults may perceive it as a form of monitoring rather than a resource for self-management [25, 26]. In our study, this uncertainty was amplified by the device’s passive design; because participants could not see how the system worked, gaps in understanding were filled with mistrust (*that app became the carer*). This aligns with studies showing that when older people are excluded from early design conversations, technologies often fail to reflect their expectations or mental models and are subsequently rejected [27].

The second theme — centred on mistrust and concerns about privacy — emerged with unusual intensity. Although scepticism toward digital systems is well documented among some older adults, the social environment of 2021–2022 magnified these apprehensions. Two contextual factors were particularly influential: first, the study involved household energy data during a period in which UK energy prices were reaching unprecedented levels; and second, older adults were simultaneously exposed to frequent warnings about digital, telephone, and energy-related scams. Within this landscape, questions such as *Who will see my* data?, *Will this affect my bill?* or *could someone tell when I’m away*? were entirely reasonable. Research on fraud awareness in later life indicates that when scam risk feels high, unsolicited contact of any form — including outreach from legitimate studies — is readily interpreted as unsafe [28, 29]. This was reflected in recruiters’ experiences, encapsulated in the sentiment that “the phone is no longer a safe place.”

For research that depends on domestic data sources such as energy-use patterns, smart-home devices, or telecare systems, standard research safeguards (ethics approval, confidentiality assurances) may therefore be insufficient. Participants’ concerns extended beyond who held the data to what could be inferred from it — whether the system could identify when they were asleep, in the bathroom, or out of the house. Such responses mirror findings from studies on privacy attitudes in later life, which consistently identify a substantial subgroup of older adults unwilling to exchange household privacy for potential technological benefit [30].

A further element within the findings was a clear lack of motivation to take part. Responses such as *No*,* I’m not interested* and *I’m not a little old man*, which were reported by a HSCW, reflected more than simple disinterest; they spoke to a desire to avoid being categorised as frail [31] and to a sense that the technology offered little practical value. When a digital tool is unfamiliar, difficult to picture, or potentially linked to financial implications, older adults tend to prioritise more immediate and concrete needs [26, 28]. In this study, participants were being asked to trust a system linked to energy data during a period of intense financial strain, without tangible short-term benefit.

Evidence from digital-health interventions indicates that approaches which allow older adults to see, touch, and trial a device — through demonstrations, hands-on sessions, or simple visual explainers — can help reduce uncertainty and strengthen understanding [32–34]. Several participants perceived the study as surveillance rather than self-management. Incorporating an in-person co-design phase with older adults and carers at community centres or in homes, despite challenges posed by pandemic restrictions, might have mitigated these concerns by shaping the study’s framing before recruitment.

The findings revealed that decisions were often taken at household level. Even when older adults expressed curiosity, sons, daughters, or carers sometimes intervened. This aligns with literature on ageing in place, which shows that families frequently act as risk mediators — interpreting remote monitoring either as an implicit judgement on the adequacy of family care or as a precursor to greater involvement from formal services. Trusted intermediaries such as community gatekeepers, GPs, or voluntary-sector workers can help mitigate these concerns by endorsing a study [35], yet our recruitment was restricted and relied largely on online platforms and telephone-first contact. A more participatory strategy — engaging through community hubs, faith organisations, or carer groups — might have activated those local trust networks earlier.

One of the more distinctive insights from this study is the way financial insecurity influenced perceptions of research legitimacy. Several older adults had deliberately reduced electricity use and struggled to see how an energy-based technology could operate in their circumstances or feared it would expose them to scrutiny. Research on smart-meter acceptance suggests that vulnerable consumers can associate the term “smart” with heightened financial risk, increased surveillance, and the potential for higher energy costs, rather than with promised efficiencies or savings [36, 37]. Few studies on ageing and technology address this dynamic, primarily because research is typically conducted outside periods of significant energy-price volatility. The present data suggest that during such periods, recruitment materials should be especially clear and incorporate considerations of additional risks that reflect the prevailing economic, environmental, and lived-experience challenges faced by potential participants.

Building on existing recommendations [38–40], we outline additional strategies tailored specifically to energy-linked and home-monitoring contexts — approaches that extend beyond general good practice and address the particular trust and contextual barriers observed in this study. *Co-empower through design reframing*: Co-creation should not only involve older adults in interface design but also in *narrative framing*, while ensuring that the framing stays within the ethical framework, this may include piloting the information sheets and recruitment materials. *Use trusted and hyper-local messengers*: While prior studies highlight the role of primary care and community partners, our findings show that trust is strongest when contact comes from familiar local figures. Credibility increased when the approach was made by a named person from the primary care practice or community organisation known to participants, rather than through generic institutional emails or unknown phone numbers. *Demystify the technology through ‘energy-literacy’ demonstrations*: Existing research calls for hands-on demos; we add that in energy-linked studies, researchers must go further — actively bridging energy literacy *and* health literacy. Demonstrations that show how a simple change in kettle or lighting use generates a signal can transform perceptions from “surveillance” to “sensing routine,” restoring a sense of control. *Layer and localise recruitment channels*: Beyond multi-modal communication (online platforms, phone, letter), our data suggests - sequencing these channels over time — starting with a familiar community touchpoint, followed by direct contact from the study team. This layered approach builds recognition and legitimacy incrementally. *Design for relational time*: Recruitment among digitally cautious older adults cannot be transactional. Trust requires multiple, low-pressure contacts — time for reflection, discussion with family, and reassurance. Embedding this relational lag within recruitment timelines should be treated as a methodological necessity, not a contingency. Future research could build on these findings by testing recruitment approaches that address the barriers identified, including improving understanding of the technology and responding to concerns about privacy and data use. Examining whether such strategies lead to increased enrolment—particularly in contexts shaped by pandemic recovery and cost-of-living pressures—would help clarify which factors are most influential in supporting participation among under-represented older adults.

### Limitations

This evaluation reflects the perspectives of a relatively small group of health and social care workers involved in recruitment, alongside community gatekeepers and carers of older adults who declined participation (*n* = 10 in total). Although the sample size was limited, the discussions were detailed and iterative, enabling a deeper exploration of participants’ experiences and reasoning than a larger, more superficial dataset might have allowed. Insights were drawn across multiple data sources—including workshop discussions, individual consultations, and recruiters’ reflective notes to support a coherent and credible analysis.

Although four individuals initially agreed to participate, the study did not progress to data collection due to insufficient enrolment. As a result, no data were collected from these participants, and their views on participation were not explored. The analysis is therefore based on the perspectives of those involved in the recruitment process rather than enrolled participants themselves. This limits the ability to distinguish factors specific to non-participation from those that may also be present among individuals who chose to enrol — and crucially, how those individuals navigated or overcame such factors in deciding to take part. This should be considered when interpreting the findings. Future studies could usefully explore not only the barriers older adults face in engaging with digital health research, but also the factors that enable some to participate despite these challenges.

While approximately 150 potential participants were documented as approached, recruitment activity extended beyond formally recorded contacts. Recruiters indicated that additional informal and telephone-based outreach may not have been systematically recorded, suggesting that the overall level of recruitment effort was likely higher than documented. This further highlights the challenges of engaging this group during a period characterised by heightened digital mistrust.

Finally, the study was context-specific conducted in a single UK city during a period marked by prolonged COVID-19 recovery and significant increases in energy costs. Transferability to other settings should therefore be considered in light of these conditions. Nonetheless, the consistency of themes across data sources, alongside their alignment with existing research on digital exclusion and technology adoption among older adults, supports the overall credibility of the findings.

## Conclusions

Recruiting housebound older adults in an inner-city context to a study that relied on energy-use data proved substantially more complex than expected during the combined pressures of COVID-19 recovery and the cost-of-living crisis. The challenge did not stem from disinterest in health, but from the interplay of surveillance concerns, financial insecurity, limited digital confidence, and family-level gatekeeping. Within these conditions, telephone-first recruitment and online platform approaches were largely ineffective. Studies involving smart-home sensors, smart-meter data or other forms of domestic-data monitoring should view recruitment as a relational process grounded in trust. This means co-designing research recruitment materials with older adults and carers, working through locally recognised intermediaries, and allowing time for familiarisation and reassurance. Approaches built on these principles will be better positioned to engage those older adults who are most vulnerable to digital exclusion — the very individuals for whom supportive technologies are often intended.

## Supplementary Information

Supplementary Material 1.

## Data Availability

The qualitative data (workshop notes, interview transcripts, field notes) contain potentially identifying information from a small group of participants and are not publicly available to protect confidentiality. De-identified excerpts are available from the corresponding author on reasonable request.
