# Supplementary material for: ‘It felt like surveillance, not support’: understanding barriers to enrolling older adults in an energy-linked digital health study during pandemic recovery and the cost-of-living crisis
Source: BMC Geriatr. 2026 May 28;26:1039. doi: 10.1186/s12877-026-07665-7 (PMC13463524; doi:10.1186/s12877-026-07665-7)
Supplement: Supplementary file 1 [file 12877_2026_7665_MOESM1_ESM.docx]

**Title: “It felt like surveillance, not support”: challenges in recruiting older adults to an energy-linked digital health study during COVID-19 recovery and the UK cost-of-living crisis**

**Qualitative Interview Topic Guide**

A qualitative design was deemed appropriate to explore experiences of recruitment to an energy-linked digital health study involving older adults and their families. Semi-structured interviews and a stakeholder workshop were conducted to allow participants to describe their experiences in their own words, while ensuring consistency across sessions. The sessions focused on the following core areas:

**1. Barriers to recruitment**

Participants were asked to reflect on their experiences of the recruitment process and any challenges encountered. Example prompts included:

- In your experience, what were the main barriers to recruitment for this project?
- What aspects of the study or recruitment approach made participation difficult or created uncertainty?
- Were there any concerns related to technology, data, privacy, or trust?
- Did the wider context (e.g. COVID-19 recovery, cost-of-living pressures, health or caring responsibilities) influence engagement?

**2. Reasons for non-participation**

Participants were asked to reflect on instances where potential participants or family members declined participation, based on their experiences as recruiters or carers. Example prompts included:

- What reasons were given by potential participants, or by family members, for deciding not to take part or not to support participation?
- What concerns or uncertainties appeared to influence these decisions?
- How did perceptions of monitoring, surveillance, or data sharing shape responses to the study?
- Were there particular aspects of the study that were perceived as unsuitable or inappropriate?

**3. Alternative recruitment approaches**

Participants were invited to offer reflections on how recruitment could have been improved. Example prompts included:

- Based on your experience, what alternative approaches to recruitment do you think might have been more effective or acceptable?
- What would have made participation feel safer, clearer, or more supportive?
- Who do you think older adults and their families would trust to introduce studies like this?
- What advice would you give researchers planning similar digital health studies with older adults?

Interviews were flexible and responsive, with prompts adapted to participants’ experiences while retaining coverage of the core topic areas. Follow-up questions were used to clarify meanings and explore issues raised by participants in greater depth.
